# Early myocardial dysfunction in streptozotocin-induced diabetic mice: a study using in vivo magnetic resonance imaging (MRI)

**DOI:** 10.1186/1475-2840-6-6

**Published:** 2007-02-19

**Authors:** Xichun Yu, Yasvir A Tesiram, Rheal A Towner, Andrew Abbott, Eugene Patterson, Shijun Huang, Marion W Garrett, Suresh Chandrasekaran, Satoshi Matsuzaki, Luke I Szweda, Brian E Gordon, David C Kem

**Affiliations:** 1 Department of Medicine, University of Oklahoma Health Sciences Center & VAMC, Oklahoma City, OK 73104, USA; 2 Cardiac Arrhythmia Research Institute, University of Oklahoma Health Sciences Center, Oklahoma City, OK 73104, USA; 3 Small Animal MRI Core Facility, Oklahoma Medical Research Foundation, Oklahoma City, OK 73104, USA; 4 Free Radical Biology and Aging Research Program, Oklahoma Medical Research Foundation, Oklahoma City, OK 73104, USA; 5 Laboratory Animal Resource Center, Oklahoma Medical Research Foundation, Oklahoma City, OK 73104, USA

## Abstract

**Background:**

Diabetes is associated with a cardiomyopathy that is independent of coronary artery disease or hypertension. In the present study we used in vivo magnetic resonance imaging (MRI) and echocardiographic techniques to examine and characterize early changes in myocardial function in a mouse model of type 1 diabetes.

**Methods:**

Diabetes was induced in 8-week old C57BL/6 mice with two intraperitoneal injections of streptozotocin. The blood glucose levels were maintained at 19–25 mmol/l using intermittent low dosages of long acting insulin glargine. MRI and echocardiography were performed at 4 weeks of diabetes (age of 12 weeks) in diabetic mice and age-matched controls.

**Results:**

After 4 weeks of hyperglycemia one marker of mitochondrial function, NADH oxidase activity, was decreased to 50% of control animals. MRI studies of diabetic mice at 4 weeks demonstrated significant deficits in myocardial morphology and functionality including: a decreased left ventricular (LV) wall thickness, an increased LV end-systolic diameter and volume, a diminished LV ejection fraction and cardiac output, a decreased LV circumferential shortening, and decreased LV peak ejection and filling rates. M-mode echocardiographic and Doppler flow studies of diabetic mice at 4 weeks showed a decreased wall thickening and increased E/A ratio, supporting both systolic and diastolic dysfunction.

**Conclusion:**

Our study demonstrates that MRI interrogation can identify the onset of diabetic cardiomyopathy in mice with its impaired functional capacity and altered morphology. The MRI technique will lend itself to repetitive study of early changes in cardiac function in small animal models of diabetic cardiomyopathy.

## Background

The existence of diabetic cardiomyopathy, a myocardial disease associated with diabetes in the absence of coronary artery disease, hypertension or any other known cardiac disease, is supported by evidence accumulated from a large and expanding literature [1-4]. Abnormalities in cardiac function and structure in diabetic subjects have been demonstrated in animal [5-8] and human studies [9-12]. The pathogenesis of this ventricular dysfunction remains unknown, but includes impaired metabolism, myocardial fibrosis, small vessel disease and autonomic neuropathy [1-4].

Mice are increasingly used in diabetes research mainly because of the ability to create genetically engineered mice for investigation of the molecular mechanisms underlying cardiovascular complications [13-19]. Chemical induction of insulin deficiency by a cytotoxic agent for pancreatic beta cells, streptozotocin (STZ), produces a well-characterized model of type 1 diabetes [20]. This model allows more accurate timing of selected metabolic events and correlation with their pathophysiologic consequences. Assessment of cardiac function in mouse models of both type 1 and type 2 diabetes has relied on conventional echocardiography [15-17,19], invasive in vivo catheterization [18,19,21], or ex vivo [14,22,23] techniques with their associated restrictions upon accuracy or serial studies in timed experiments. The noninvasive magnetic resonance imaging (MRI) techniques provide a 3-dimensional representation of cardiac structure and function, and have proven to be a reliable and reproducible means of studying in vivo cardiac morphology and function in mice under physiological conditions [24-28]. Only recently, the use of cardiac MRI has been reported for assessment of the cardiomyopathic changes in type 2 diabetic mice [29].

The purpose of the present study was to use MRI techniques to examine the early onset of myocardial dysfunction in STZ-induced diabetic mice. These studies can then be applied to interventions that may alter or prevent the onset of this condition using the advantage of repetitive examinations over selected periods of time.

## Methods

### Mice

6–8 week old C57BL/6 female mice were obtained from Harlan (Indianapolis, IN) and housed at the MRI animal facility. All animals had free access to standard laboratory chow and water. Animal protocols were approved by the local Institutional Animal Care and Use Committee.

### Induction of diabetes

Mice were injected intraperitoneally with 100 mg/kg of STZ (in 0.05 mol/l citrate buffer, pH 4.5, Sigma, St. Louis, MO) or vehicle once a day for two consecutive days. Tail blood glucose (TBG) levels were measured using a glucose oxidase test strip (Lifescan, Milpitas, CA). Animals were considered to be diabetic if their TBG is > 13 mmol/l. The blood glucose levels and body weight of all mice were monitored biweekly. When their TBG is > 22 mmol/l, they were given intermittent low dosages of long acting insulin glargine [30] to maintain blood glucose at 19–25 mmol/l. Mitochondrial NADH oxidase activity was determined in ventricular tissues from diabetic mice, and MRI studies were performed on diabetic mice at 4 weeks. Age matched controls were included for comparisons.

### Mouse preparation

For MRI studies, mice were anesthetized with inhaled isoflurane (1.5% at 1 L/min oxygen flow) via a nose cone. During the experiment, the mouse was positioned supine on a nonmagnetic warming pad to maintain constant body temperature throughout the MR study.

### In vivo MRI

Experiments were performed on a 7 Tesla MR scanner (Bruker BioSpin, Ettlingen, Germany). For cardiac imaging the gradient echo sequence [31] with *t*_e _= 4.0 ms, *t*_r _= 100 ms, and a flip angle, *θ *= 85°–90° was used for all experiments. The heart was initially positioned by measurement to the center of the magnet and then further set by observing the position using fast imaging with steady state precession (FISP) [32] as the animal was moved. Once the heart was in the iso-center a gradient echo sequence with *t*_e _= 4.0 ms and *t*_r _= 50 ms was used to acquire single slice images in all three (axial, sagittal and coronal) planes. The slice position for the cine was set using this pilot scan. For cine and volume imaging a field of view, *FOV *= 2.56 × 2.56 cm was chosen and 256 each of phase encode and read steps were used to resolve the spatial distribution of excited spins. The image was made after zero filling in each direction to 512 × 512. Window functions were not used and some minor image quality enhancement may be achieved if window functions are used. For our purpose the signal contrast of the myocardium and "white" blood was sufficient to allow calculation of the relevant parameters.

Spectrometer triggering with combined respiratory and ECG triggers was used in all experiments. Thus, all MRI data are adjusted to accommodate for rate changes and the data are reflective of the ambient cardiac function and rate. The cardiac period, *P*, of mice used in these studies ranged from ~120–180 ms, and a respiratory rate of ~18–30 bpm. Trigger delays, *t*_d_, were incremented at the spectrometer hardware in a chosen number of steps, *n*_steps_, (*n*_steps _= 10) according to *t*_d _= (*P*-(*P*/*n*_steps_))/*P*, where period is the time between the peaks of the *R *wave in the ECG trace. The images at each increment of the period were combined and displayed by cine in the mid-ventricular short axis slice. These provided data for assessment of left ventricular (LV) systolic and diastolic dynamics, and for calculations of fractional circumferential shortening and wall thickening after measuring the endocardial circumferential length and myocardial wall thickness at end diastole and end systole.

For volume calculations, a new set of images were collected with the slice position advanced by the slice thickness of 1.0 mm. In order to reduce the time that multi-slice experiments fit in to the repetition time (Tr) used for single slice experiments, the slice thickness was increased to 1.25 mm. Thus, in this case, the resolution in the slice shifting is compromised by 0.25 mm at most, and contributes an error of about 0.1% in total volume calculations compared with a 1 mm slice thickness. The total blood volume at end diastole and end systole was estimated by taking the sum of all cavity slice volumes assuming a uniform thickness of excitation across a chosen slice at the two trigger points.

### MRI analysis

For LV blood volume measurements, endocardial borders were manually delineated. An independent observer was used whenever a questionable or indistinct image was encountered. These measurements rarely changed the summed data obtained from a given data acquisition. The end-diastolic volume (EDV) and end-systolic volume (ESV) were calculated as the sum of all cavity slice volumes at end diastole and end systole. For assessment of LV systolic and diastolic dynamics, the cavity slice volume was measured in all acquired images and was plotted against the time from onset of the QRS trigger, and a volume-time curve was established. Peak ejection rate and peak filling rate were calculated from the maximum slopes of the LV ejection and filling curve [33].

### Echocardiography

M-mode/Doppler echocardiography was performed using a 13 MHz 15L8 probe and an Acuson Sequoia 256 echocardiography system (Acuson, Mountain View, CA). The wall and cavity dimensions during diastole and systole were determined from long-axis views of the left ventricle [34]. The mitral flow velocities were assessed by Doppler, and the E wave and A wave were measured to provide an estimate of diastolic function [35].

### Mitochondrial function

Frozen and thawed mitoplasts from hearts were diluted into 10 mmol/l 3-(N-morpholino)propane-sulfonic acid (MOPS), 25 mmol/l KCl at pH 7.4 to a final concentration of 50 μg/ml [36]. NADH oxidase activity was measured as the rate of NADH disappearance at 340 nm upon addition 200 μM NADH [36]. Citrate synthase activity was monitored by detection of 5,5'-dithiobis(2-nitrobenzoic acid) (DTNB) reactive CoASH (412 nm, ε = 13,600) upon addition of 0.1 mmol/l DTNB, 0.3 mmol/l acetyl CoA, and 0.5 mmol/l oxaloacetate to frozen and thawed mitoplasts (20 μg/ml) [37]. All enzyme assays were performed at room temperature.

### Statistical analysis

Data are expressed as mean ± SEM. An unpaired 2-tailed Student *t *test was used to make comparisons between control and diabetic groups. Significance was ascribed to *P *values < 0.05.

## Results

### Effects of STZ on blood glucose and body weight

**Figure 1:**
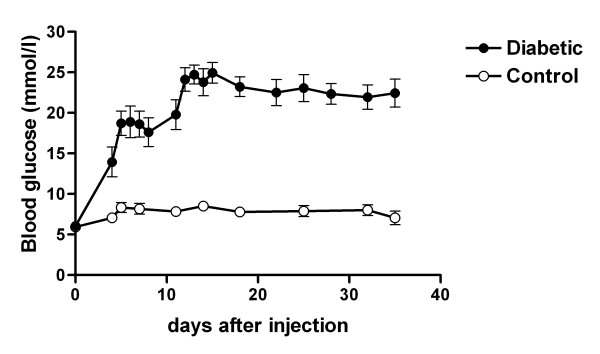
**Blood glucose levels in control and diabetic mice**. Mice were treated with streptozotocin (STZ) or citrate buffer on days 0 and 1. The onset of hyperglycemia occurred within one week after STZ injection. Mice were given low dosages of long acting insulin to maintain blood glucose at 19–25 mmol/l. Values are mean ± SEM. Control, n = 12; diabetic, n = 8.

The mice generally developed significant elevation of blood glucose within a week following STZ injection (Figure 1). The STZ-treated mice showed blood glucose levels ranging from 17 to over 27 mmol/l as compared with 5.5–8.3 mmol/l for citrate-treated control mice. Diabetic mice with blood glucose levels of > 22 mmol/l were given low dosages of long acting insulin (1–1.5 IU s.c.) daily to maintain their blood glucose at 19–25 mmol/l. After 4 weeks of diabetes, the diabetic mice had a 13% lower body weight and an 11% lower heart weight than non-diabetic control mice. The ratio of heart weight to body weight was not significantly changed (Table 1).

**Table 1:** Body weight and heart weight data from control and diabetic mice.

	Control (n = 8)	Diabetic (n = 12)	*P *value
Body weight (g)	20.2 ± 0.4	17.6 ± 0.6	< 0.01
Heart weight (mg)	86.8 ± 1.2	77.3 ± 1.7	< 0.001
HW/BW ratio (mg/g)	4.3 ± 0.1	4.4 ± 0.1	NS

Values are mean ± SEM. HW/BW, heart weight to body weight ratio.

### Myocardial morphology and function in diabetic mice

**Figure 2:**
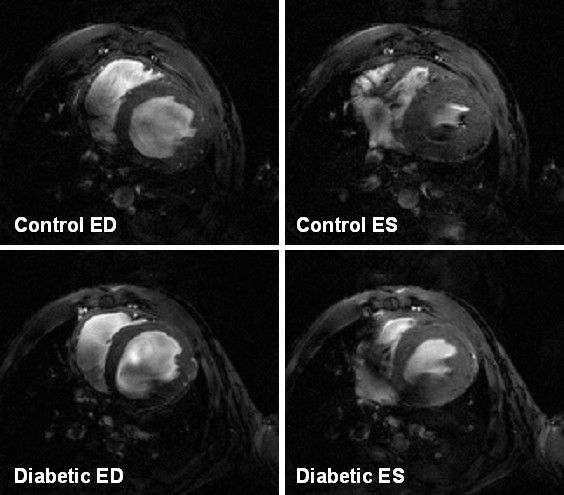
**End-diastolic (left) and end-systolic (right) MR images in a mid-ventricular slice from a control (top) and diabetic (bottom) mouse**. The LV diameters for the control and diabetic mouse were similar at end diastole (ED), but apparently increased at end systole (ES) for the diabetic mouse heart compared with control. The wall thickness at both end diastole and end systole in the diabetic mouse heart was decreased compared with control.

The animals tolerated the anesthesia and procedure without difficulty. There were no post study deaths. The fast-gradient echo MR technique provides sufficient contrast between blood and myocardium to allow clear delineation of epicardial and endocardial borders (Figure 2). LV diameters and wall thickness were measured in mid-ventricular short-axis slices at end diastole and end systole. After 4 weeks of diabetes, no significant change of endocardial diameters at end diastole was observed (3.6 ± 0.1 vs. 3.4 ± 0.1 mm). However, endocardial diameters were significantly increased at end systole (2.4 ± 0.1 vs. 1.8 ± 0.1 mm, *P *< 0.001). Diabetic mice also showed significant thinning of the LV wall compared to control animals both at end diastole (0.85 ± 0.03 vs. 1.01 ± 0.05 mm, *P *< 0.01) and at end systole (1.19 ± 0.05 vs. 1.51 ± 0.06 mm, *P *< 0.001) (Table 2 & Figure 2).

**Table 2:** Comparison of MRI-derived LV diameter and wall thickness in control and diabetic mice.

	Control (n = 8)	Diabetic (n = 12)	*P *value
Internal diameter, diastole (mm)	3.4 ± 0.1	3.6 ± 0.1	NS
Internal diameter, systole (mm)	1.8 ± 0.1	2.4 ± 0.1	< 0.001
Wall thickness, diastole (mm)	1.01 ± 0.05	0.85 ± 0.03	< 0.01
Wall thickness, systole (mm)	1.51 ± 0.06	1.19 ± 0.05	< 0.001

Values are mean ± SEM.

The heart rates were similar between diabetic and control mice (446 ± 11 vs. 470 ± 11 bpm). To determine the effects of diabetes on LV systolic function, the LV blood volumes were measured from multiple short-axis slices that cover the whole heart at end diastole and end systole. The EDV was not significantly changed after 4 weeks of diabetes (44.8 ± 1.0 vs. 46.0 ± 1.1 μl). The ESV, on the other hand, was increased by 23%. Therefore, a significantly reduced myocardial contractile function was observed in diabetic mice. The stroke volume, ejection fraction and cardiac output were decreased by 20%, 18% and 16%, respectively. A similar decrease in LV fractional circumferential shortening was also seen in diabetic mice (Table 3).

**Table 3:** Comparison of MRI-derived LV volume and systolic function between control and diabetic mice.

	Control (n = 8)	Diabetic (n = 12)	*P *value
Heart Rate (bpm)	470 ± 11	446 ± 11	NS
End-diastolic volume (μl)	46.0 ± 1.1	44.8 ± 1.0	NS
End-systolic volume (μl)	18.3 ± 0.5	22.6 ± 0.5	< 0.001
Stroke volume (μl)	27.8 ± 0.7	22.3 ± 0.8	< 0.001
Ejection fraction (%)	60.3 ± 0.6	49.6 ± 1.3	< 0.001
Cardiac output (ml/min)	13.1 ± 0.4	9.9 ± 0.5	< 0.05
Fractional shortening (%)	37.7 ± 1.4	24.2 ± 1.6	< 0.001

Values are mean ± SEM.

**Figure 3:**
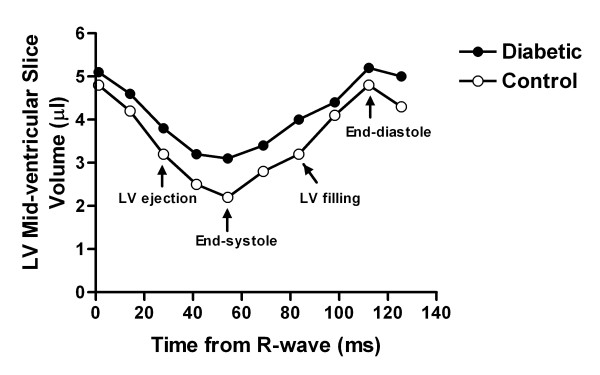
**LV volume-time curves from a representative control and diabetic mouse**. The LV cavity slice volume in a mid-ventricular short-axis cine image is plotted against time of acquisition within the cardiac cycle. The difference of end-systolic volumes between control and diabetic mouse is apparent, while the end-diastolic volumes are similar. The maximum ejection rate and maximum filling rate calculated from these curves both are decreased in diabetic mice.

**Figure 4:**
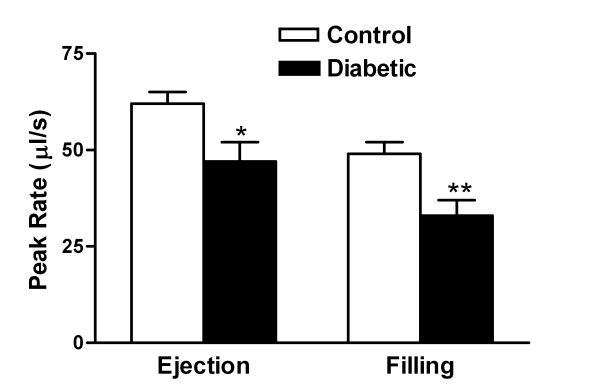
**Peak LV ejection and filling rates calculated from the maximum slopes of the volume-time curves for control and diabetic mice**. These data were obtained from the LV volume-time curve for each mouse, as shown for a single mouse in Figure 3. Values are mean ± SEM. *P < 0.05 and **P < 0.01 vs. control, n = 8.

To assess LV systolic and diastolic dynamics, peak ejection rate and peak filling rate were calculated from the systolic and diastolic limbs of the volume-time curve (Figure 3). In diabetic mice compared to controls, there was a significantly reduced maximum LV ejection rate (47 ± 5 vs. 62 ± 3 μl/s, n = 8, *P *< 0.05) and filling rate (33 ± 4 vs. 49 ± 3 μl/s, n = 8, *P *< 0.01) indicating a decrease of both contraction and relaxation (Figures 3 &4).

**Figure 5:**
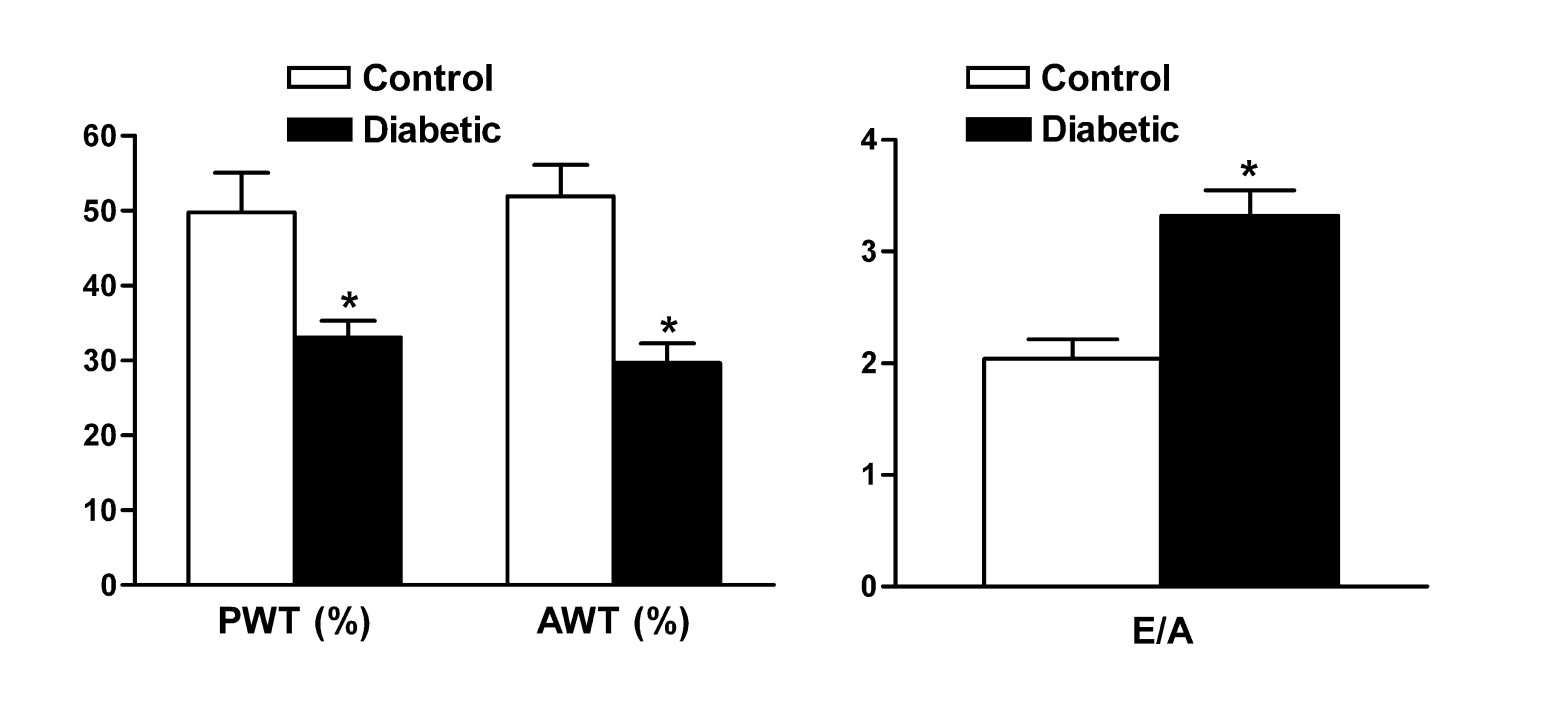
**M-mode echocardiographic and Doppler flow analysis on control and diabetic mice**. Cardiac performance was examined 4 weeks after onset of hyperglycemia. PWT, posterior wall thickening; AWT, anterior wall thickening; E/A, E to A ratio. Values are mean ± SEM. Control, n = 10; diabetic, n = 23. *P < 0.001 vs. control.

To compare the MRI techniques with conventional echocardiography, M-mode echocardiographic and Doppler flow studies were performed on control and 4-wk STZ-diabetic mice. The diabetic mice showed a decreased wall thickening and increased E/A ratio, indicating that STZ-induced diabetes affects both systolic and diastolic function (Figure 5).

### Effect of diabetes on mitochondrial NADH oxidase and citrate synthase activity

**Figure 6:**
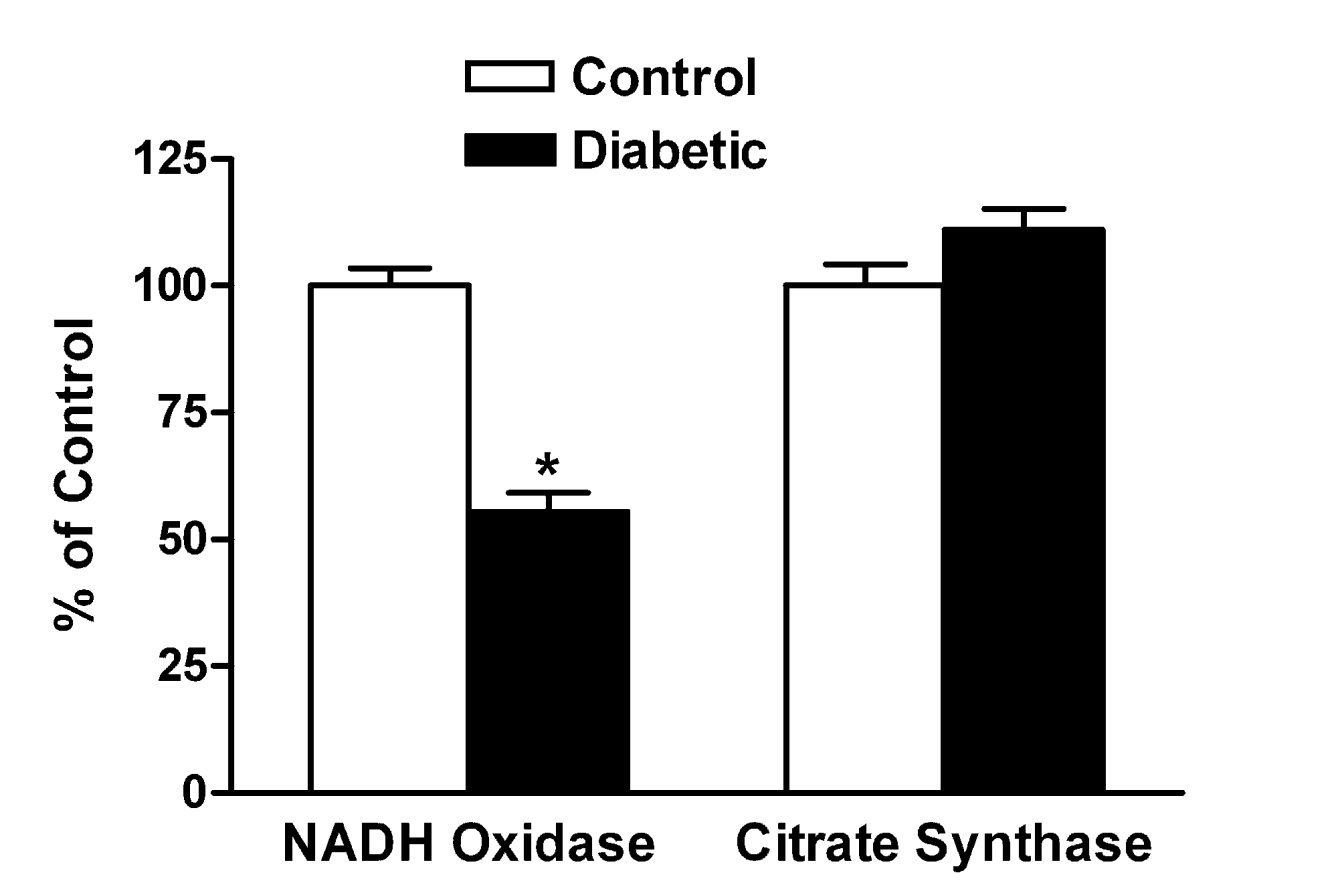
**Effect of diabetes on mitochondrial NADH oxidase and citrate synthase activity**. Mitoplasts were prepared from frozen hearts (n = 5) isolated from control and 4-week diabetic mice. Following solubilization of the mitoplasts, NADH oxidase and citrate synthase activity were measured as described. Values represent the mean ± SD. *P < 0.001 vs. control.

To begin to assess the effects of diabetes on mitochondrial function, NADH oxidase activity, a measure of overall electron transport, was determined in mitoplasts prepared from hearts isolated from control and diabetic (4 weeks) mice. As shown in Figure 6, diabetes resulted in a decline in NADH oxidase activity of approximately 50% relative to control values. This was not due to an overall decline in mitochondrial protein content given that the yield of mitoplasts (not shown) and the activity of the Krebs cycle enzyme citrate synthase (Figure 6) did not vary between control and diabetic hearts. Therefore, this parameter of mitochondrial electron transport activity was severely compromised within 4 weeks of the onset of marked hyperglycemia. In contrast to the nearly 50% decline in cardiac NADH-linked electron transport chain activity (NADH oxidase) induced by diabetes, complex I activity declined 21.2% (*P *value ≤ 0.003). Complex I is the rate limiting step in electron transport chain. Therefore this decrement is, in part, responsible for the observed loss in NADH oxidase activity. Other deficits that contribute to the diabetes-induced declines in electron transport are under investigation.

## Discussion

It has been difficult in obtaining sequential analyses of early diabetic cardiomyopathy in humans, and even in small animal models. The cardiac phenotype of diabetic mice has been examined using in vivo and ex vivo techniques in both type 1 and type 2 diabetic mouse models [14-19,21-23]. In vivo evidence for cardiac dysfunction in diabetic mice is commonly obtained from echocardiography [15-17] and/or invasive catheterization [18,21].

Transthoracic echocardiography is a noninvasive method for in vivo assessment of cardiac function. It is readily available and very useful for serial studies on age-dependent cardiac changes [38,39]. M-mode echocardiography provides indices of LV systolic function and calculated LV mass, while Doppler measurements of transmitral flow provide an index of diastolic function [26,40]. Systolic and diastolic dysfunction has been demonstrated in diabetic mice [15-17]. However, these animals were either severely diabetic (blood glucose of > 33 mmol/l for 3 weeks) or chronically diabetic (12 weeks). In a study of 4-week STZ-diabetic mice, echocardiography did not reveal a significantly reduced systolic function [19]. This may relate to the less sensitive techniques used for systolic function evaluation. Additionally, methods for measurement of cardiac mass and volume generally depend on use of linear measurements and calculation of mass and volume based on geometric assumptions that may or may not be accurate for that organ.

MRI provides accurate, reproducible, noninvasive 3-dimensional representations of cardiac structure and function. The MRI techniques have been adapted and validated to assess mouse cardiac function in vivo [24-28]. Unlike echocardiography, the 3-dimensional MR images also provide an accurate analysis of morphological changes without requiring geometric assumptions [26]. Our studies using this technique clearly demonstrated that STZ-diabetic mice had ventricular wall thinning, increased end-systolic diameter and volume, diminished ejection fraction, decreased circumferential shortening, and decreased peak ejection and filling rates. These changes are consistent with those described using other in vivo techniques at various stages in development of diabetic cardiomyopathy [15-18,21]. The MRI interrogation of myocardial performance appears to offer the sensitivity needed for the early detection of physiological events that correlate with the onset of metabolic and other pathophysiologic mechanisms that are etiologic in diabetic cardiomyopathy.

In preliminary studies, we examined one marker of the onset of mitochondrial dysfunction (NADH oxidase activity) and demonstrated this parameter was decreased by 50% at 4 weeks. This translates into a significant deficiency of mitochondrial electron transport and serves as one of several indicators of an impaired cardiac energy state in early diabetes. These data help confirm a metabolic basis for the observed impairment of cardiac function using MRI interrogation. Mitochondrial dysfunction has been reported in the diabetic heart [41,42]. Such reduced energy stores would lead to subsequent myocardial dysfunction. Since mitochondrial ATP synthesis is the major source of energy in cardiomyocytes, repetitive measurement of this parameter would be of value. Future work must establish precise molecular mechanisms by which diabetes leads to declines in mitochondrial function and determine whether these decrements are of sufficient magnitude to limit the supply of energy for cardiac function.

Recently, MR spectroscopy has been used in mice to examine myocardial energy metabolism [27,43]. ^31^P spectra provide the only *noninvasive *method to quantitate myocardial high-energy phosphates (ATP, phosphocreatine). This novel technique should prove complementary to standard MRI interrogation to study the contribution of altered energy metabolism to the development of diabetic cardiomyopathy. The major drawback at present is the design of appropriate small coils that will permit accurate localization of data to the myocardium separate from the underlying blood.

Despite its advantages, MRI has limitations. It is costly, time consuming, has blood flow and motion generated artifacts, and has limited availability. Application of sensitive, discriminate software to minimize manual determination of selected cardiac parameters such as cardiac borders in repetitive slices will decrease the time required for analysis. We have found that the time required for positioning and acquisition of the relevant data was significantly decreased with experience of the personnel.

## Conclusion

In summary, we have implemented a noninvasive MRI technique that was used for the first time to detect and characterize the early onset of myocardial dysfunction in a mouse model of type 1 diabetes. This provides a powerful approach to examining early cardiac dysfunction in vivo in mice with transgenic manipulation or pharmacological interventions that will ultimately lead to a better understanding of the pathophysiology and to reduction of the incidence of diabetic cardiomyopathy in humans.

## Competing interests

The author(s) declare that they have no competing interests.

## Authors' contributions

XY and DCK conceived the study, participated in its design and coordination, performed and analyzed the data with statistical analysis and drafted the manuscript. RAT participated in the design of the study, provided expertise and oversight in the MRI procedures and helped with manuscript preparation. YAT and AA carried out the MRI data acquisition and helped with data analysis and manuscript preparation. SH and MWG carried out the diabetes induction and mouse maintenance. EP and SC carried out the echocardiography and data collection, and helped with data analysis. SM performed the mitochondrial assay, and LIS helped with data interpretation and manuscript preparation. BEG supervised the mouse maintenance and assisted with data analysis. All authors read and approved the final manuscript.

## References

[B1] Fang ZY, Prins JB, Marwick TH (2004). Diabetic cardiomyopathy: evidence, mechanisms, and therapeutic implications. Endocr Rev.

[B2] Hayat SA, Patel B, Khattar RS, Malik RA (2004). Diabetic cardiomyopathy: mechanisms, diagnosis and treatment. Clin Sci (Lond).

[B3] Bell DS (2003). Diabetic cardiomyopathy. Diabetes Care.

[B4] Regan TJ, Ahmed S, Haider B, Moschos C, Weisse A (1994). Diabetic cardiomyopathy: experimental and clinical observations. N J Med.

[B5] Fein FS, Kornstein LB, Strobeck JE, Capasso JM, Sonnenblick EH (1980). Altered myocardial mechanics in diabetic rats. Circ Res.

[B6] Travers KE, Perreault-Micale CL, Hampton T, Katz SE, Morgan JP, Douglas PS, Joffe (1999). Abnormal cardiac function in the streptozotocin-induced non-insulin-dependent diabetic rat: noninvasive assessment with doppler echocardiography and contribution of the nitric oxide pathway. J Am Coll Cardiol.

[B7] Hoit BD, Castro C, Bultron G, Knight S, Matlib MA (1999). Noninvasive evaluation of cardiac dysfunction by echocardiography in streptozotocin-induced diabetic rats. J Card Fail.

[B8] Thompson EW (1988). Structural manifestations of diabetic cardiomyopathy in the rat and its reversal by insulin treatment. Am J Anat.

[B9] Rubler S, Dlugash J, Yuceoglu YZ, Kumral T, Branwood AW, Grishman A (1972). New type of cardiomyopathy associated with diabetic glomerulosclerosis. Am J Cardiol.

[B10] Regan TJ, Lyons MM, Ahmed SS, Levinson GE, Oldewurtel HA, Ahmad MR, Haider B (1977). Evidence for cardiomyopathy in familial diabetes mellitus. J Clin Invest.

[B11] Friedman NE, Levitsky LL, Edidin DV, Vitullo DA, Lacina SJ, Chiemmongkoltip P (1982). Echocardiographic evidence for impaired myocardial performance in children with type I diabetes mellitus. Am J Med.

[B12] Nunoda S, Genda A, Sugihara N, Nakayama A, Mizuno S, Takeda R (1985). Quantitative approach to the histopathology of the biopsied right ventricular myocardium in patients with diabetes mellitus. Heart Vessels.

[B13] Leiter EH (2002). Mice with targeted gene disruptions or gene insertions for diabetes research: problems, pitfalls, and potential solutions. Diabetologia.

[B14] Trost SU, Belke DD, Bluhm WF, Meyer M, Swanson E, Dillmann WH (2002). Overexpression of the sarcoplasmic reticulum Ca(2+)-ATPase improves myocardial contractility in diabetic cardiomyopathy. Diabetes.

[B15] Suarez J, Belke DD, Gloss B, Dieterle T, McDonough PM, Kim YK, Brunton LL, Dillmann WH (2004). In vivo adenoviral transfer of sorcin reverses cardiac contractile abnormalities of diabetic cardiomyopathy. Am J Physiol Heart Circ Physiol.

[B16] Semeniuk LM, Kryski AJ, Severson DL (2002). Echocardiographic assessment of cardiac function in diabetic db/db and transgenic db/db-hGLUT4 mice. Am J Physiol Heart Circ Physiol.

[B17] Nielsen LB, Bartels ED, Bollano E (2002). Overexpression of apolipoprotein B in the heart impedes cardiac triglyceride accumulation and development of cardiac dysfunction in diabetic mice. J Biol Chem.

[B18] Kajstura J, Fiordaliso F, Andreoli AM, Li B, Chimenti S, Medow MS, Limana F, Nadal-Ginard B, Leri A, Anversa P (2001). IGF-1 overexpression inhibits the development of diabetic cardiomyopathy and angiotensin II-mediated oxidative stress. Diabetes.

[B19] Harris IS, Treskov I, Rowley MW, Heximer S, Kaltenbronn K, Finck BN, Gross RW, Kelly DP, Blumer KJ, Muslin AJ (2004). G-protein signaling participates in the development of diabetic cardiomyopathy. Diabetes.

[B20] Like AA, Rossini AA (1976). Streptozotocin-induced pancreatic insulitis: new model of diabetes mellitus. Science.

[B21] Pacher P, Liaudet L, Soriano FG, Mabley JG, Szabo E, Szabo C (2002). The role of poly(ADP-ribose) polymerase activation in the development of myocardial and endothelial dysfunction in diabetes. Diabetes.

[B22] Aasum E, Hafstad AD, Severson DL, Larsen TS (2003). Age-dependent changes in metabolism, contractile function, and ischemic sensitivity in hearts from db/db mice. Diabetes.

[B23] Belke DD, Larsen TS, Gibbs EM, Severson DL (2000). Altered metabolism causes cardiac dysfunction in perfused hearts from diabetic (db/db) mice. Am J Physiol Endocrinol Metab.

[B24] Vallee JP, Ivancevic MK, Nguyen D, Morel DR, Jaconi M (2004). Current status of cardiac MRI in small animals. Magma.

[B25] James JF, Hewett TE, Robbins J (1998). Cardiac physiology in transgenic mice. Circ Res.

[B26] Hoit BD (2001). New approaches to phenotypic analysis in adult mice. J Mol Cell Cardiol.

[B27] Chacko VP, Aresta F, Chacko SM, Weiss RG (2000). MRI/MRS assessment of in vivo murine cardiac metabolism, morphology, and function at physiological heart rates. Am J Physiol Heart Circ Physiol.

[B28] Zhou R, Pickup S, Glickson JD, Scott CH, Ferrari VA (2003). Assessment of global and regional myocardial function in the mouse using cine and tagged MRI. Magn Reson Med.

[B29] Yue P, Arai T, Terashima M, Sheikh AY, Cao F, Charo DN, Hoyt G, Robbins RC, Ashley EA, Wu J, Yang PC, Tsao PS (2006). Magnetic resonance imaging of progressive cardiomyopathic changes in the db/db mouse. Am J Physiol Heart Circ Physiol.

[B30] Schneider S, Weber R, Luippold G (2004). Blood glucose profiles in diabetic rodents using different insulin preparations. Arzneimittelforschung.

[B31] Tkach JA, Haacke EM (1988). A comparison of fast spin echo and gradient field echo sequences. Magn Reson Imaging.

[B32] Oppelt A, Graumann R, Barfuss H, Fischer H, Hartl W, Schajor W (1986). FISP: a new fast MRI sequence. Electromedica.

[B33] Wiesmann F, Ruff J, Engelhardt S, Hein L, Dienesch C, Leupold A, Illinger R, Frydrychowicz A, Hiller KH, Rommel E, Haase A, Lohse MJ, Neubauer S (2001). Dobutamine-stress magnetic resonance microimaging in mice : acute changes of cardiac geometry and function in normal and failing murine hearts. Circ Res.

[B34] Gardin JM, Siri FM, Kitsis RN, Edwards JG, Leinwand LA (1995). Echocardiographic assessment of left ventricular mass and systolic function in mice. Circ Res.

[B35] Pollick C, Hale SL, Kloner RA (1995). Echocardiographic and cardiac Doppler assessment of mice. J Am Soc Echocardiogr.

[B36] Matsuzaki S, Szweda LI (2007). Inhibition of complex I by ca(2+) reduces electron transport activity and the rate of superoxide anion production in cardiac submitochondrial particles. Biochemistry.

[B37] Nulton-Persson AC, Szweda LI (2001). Modulation of mitochondrial function by hydrogen peroxide. J Biol Chem.

[B38] Du XJ, Gao XM, Wang B, Jennings GL, Woodcock EA, Dart AM (2000). Age-dependent cardiomyopathy and heart failure phenotype in mice overexpressing beta(2)-adrenergic receptors in the heart. Cardiovasc Res.

[B39] Semeniuk LM, Severson DL, Kryski AJ, Swirp SL, Molkentin JD, Duff HJ (2003). Time-dependent systolic and diastolic function in mice overexpressing calcineurin. Am J Physiol Heart Circ Physiol.

[B40] Collins KA, Korcarz CE, Lang RM (2003). Use of echocardiography for the phenotypic assessment of genetically altered mice. Physiol Genomics.

[B41] Nishio Y, Kanazawa A, Nagai Y, Inagaki H, Kashiwagi A (2004). Regulation and role of the mitochondrial transcription factor in the diabetic rat heart. Ann N Y Acad Sci.

[B42] Shen X, Zheng S, Thongboonkerd V, Xu M, Pierce WM, Klein JB, Epstein PN (2004). Cardiac mitochondrial damage and biogenesis in a chronic model of type 1 diabetes. Am J Physiol Endocrinol Metab.

[B43] Naumova AV, Weiss RG, Chacko VP (2003). Regulation of murine myocardial energy metabolism during adrenergic stress studied by in vivo 31P NMR spectroscopy. Am J Physiol Heart Circ Physiol.

